# Single Er^3+^, Yb^3+^: KGd_3_F_10_ Nanoparticles for Nanothermometry

**DOI:** 10.3389/fchem.2021.712659

**Published:** 2021-07-21

**Authors:** Karmel de Oliveira Lima, Luiz Fernando dos Santos, Rodrigo Galvão, Antonio Claudio Tedesco, Leonardo de Souza Menezes, Rogéria Rocha Gonçalves

**Affiliations:** ^1^ Laboratório de Materiais Luminescentes Micro e Nanoestruturados—Mater Lumen, Departamento de Química, Faculdade de Filosofia, Ciências e Letras de Ribeirão Preto, Universidade de São Paulo, Ribeirão Preto, Brazil; ^2^ Departamento de Física, Universidade Federal de Pernambuco, Recife, Brazil; ^3^ Center of Nanotechnology and Tissue Engineering—Photobiology and Photomedicine Research Group, Department of Chemistry, Faculty of Philosophy, Science and Letters of Ribeirão Preto, University of São Paulo, Ribeirão Preto, Brazil

**Keywords:** KGd3F10, fluoride, lanthanides, single-nanoparticle, upconversion, nanothermometry

## Abstract

Among several optical non-contact thermometry methods, luminescence thermometry is the most versatile approach. Lanthanide-based luminescence nanothermometers may exploit not only downshifting, but also upconversion (UC) mechanisms. UC-based nanothermometers are interesting for biological applications: they efficiently convert near-infrared radiation to visible light, allowing local temperatures to be determined through spectroscopic investigation. Here, we have synthesized highly crystalline Er^3+^, Yb^3+^ co-doped upconverting KGd_3_F_10_ nanoparticles (NPs) by the EDTA-assisted hydrothermal method. We characterized the structure and morphology of the obtained NPs by transmission electron microscopy, X-ray diffraction, Raman spectroscopy, and dynamic light scattering. Nonlinear spectroscopic studies with the Er^3+^, Yb^3+^: KGd_3_F_10_ powder showed intense green and red emissions under excitation at 980 and 1,550 nm. Two- and three-photon processes were attributed to the UC mechanisms under excitation at 980 and 1,550 nm. Strong NIR emission centered at 1,530 nm occurred under low 980-nm power densities. Single NPs presented strong green and red emissions under continuous wave excitation at 975.5 nm, so we evaluated their use as primary nanothermometers by employing the Luminescence Intensity Ratio technique. We determined the temperature felt by the dried NPs by integrating the intensity ratio between the thermally coupled ^2^H_11/2_→^4^I_15/2_ and ^4^S_3/2_→^4^I_15/2_ levels of Er^3+^ ions in the colloidal phase and at the single NP level. The best thermal sensitivity of a single Er^3+^, Yb^3+^: KGd_3_F_10_ NP was 1.17% at the single NP level for the dry state at 300 K, indicating potential application of this material as accurate nanothermometer in the thermal range of biological interest. To the best of our knowledge, this is the first promising thermometry based on single KGd_3_F_10_ particles, with potential use as biomarkers in the NIR-II region.

## Introduction

In recent years, non-contact optical thermometers have played a crucial role in environments where conventional sensors are not feasible. Temperature-sensitive fluorescent nanoprobes, such as quantum dots (QDs), dyes and lanthanide-based nanoparticles (NPs) have attracted much interest in several fields due to their high sensitivity and noninvasive features. However, most QDs are cytotoxic and suffer from emission blinking, whereas dyes are easily photobleachable, and the majority of both alternatives requires ultraviolet/blue excitation (Jaque and Vetrone, 2012; Brites et al., 2016). Recently, NIR excitation/emission have started to be explored in QDs and dyes (Xu et al., 2016; Kamimura et al., 2017). In contrast with QD and dyes, lanthanide-based NPs can be excited in the most applied optical biological tissue window (*λ* ≈ 700–1700 nm) with low-power density, near-infrared (NIR) sources, which allows for deeper tissue penetration without tissue autofluorescence. Furthermore, Ln^3+^-doped materials can have relatively low cytotoxicity, narrow absorption and emission bands, and long excited-state lifetimes (Gai et al., 2013), making them interesting materials for biophotonics.

Numerous lanthanide-based materials exhibit upconversion (UC) emission process in the visible region through excitation in the NIR range via anti-Stokes shift, in which multiple photons in a sequential mode are absorbed considering the presence of excited intermediate states (Auzel, 2004). Nowadays, several works exploit this phenomenon for applications in nanoscience and nanomedicine, once such visible UC emission under NIR excitation provides the creation of multifunctional platforms in photodynamic therapy, where these materials can be used as coadjutant agents to originate a NIR-triggerable drug delivery systems. In this sense, lanthanide-based NPs have been combined with photosensitizer drug nanocarriers to activate drugs using NIR light: under NIR excitation, these luminescent systems convert the NIR to UV or visible emissions, which triggers photoreactions in the biological system (Jalani et al., 2018).

In addition, fluorescence imaging exploiting both NIR excitation and the second near-infrared (NIR-II, 1,000–1,700 nm) biological window has been widely studied for its potential use in deep tissue imaging, cancer diagnosis and treatment (Naczynski et al., 2013; Xu et al., 2019; Yu et al., 2020). NIR-to-NIR conversion provides a more efficient luminophore for bioimaging in deeper tissues because of their higher penetrability and lower radiation toxicity as compared to visible and ultraviolet radiation excitation, respectively.

The thermometric properties of luminescence materials, including the excited state lifetime (Jiang et al., 2021), emission shift (Kolesnikov et al., 2019) and luminescence intensity ratio (LIR) (Runowski et al., 2020; Galvão et al., 2021a), have been extensively studied for their non-contact operating mode, fast measurement, and high precision and resolution. Compared to other optical thermometry methods, the LIR technique can avoid the influences of spectral losses and fluctuation of excitation sources, so it is highly reliable, improves measurement accuracy, and is easy to operate (Brites et al., 2016). Among lanthanide ions, Er^3+^ dopants are usually employed for thermal sensing applications because of their two thermally coupled ^2^H_11/2_ and ^4^S_3/2_ energy levels and their low energy separation. According to the Boltzmann distribution, the small separation between the energy levels promotes ^4^S_3/2_ level depopulation toward the ^2^H_11/2_ level upon increasing temperature (Balabhadra et al., 2017).

UC processes are particularly efficient on fluoride host materials in comparison with other UC matrices, presenting good thermal and chemical stabilities (Haase and Schäfer, 2011). Nevertheless, in more recent studies, some authors have pointed out the importance of evaluating the risk to phase transformation or the effect of ion dissolution on the nanoparticle luminescence and their integrity, indicating possibility to be dependent on time, particles concentration in aqueous medium and an effective prevention using different strategies as e.g., surface functionalization (Lahtinen et al., 2017; Liu et al., 2020).

With regard to the properties of KF-GdF_3_-based systems, they are interesting luminescence hosts due to both their structural and optical properties, and they possess attractive magnetic properties owing to the intrinsic magnetic moment of Gd^3+^ ions in the matrix (Grzyb and Lis, 2009; Fedorov et al., 2011). The presence of Gd^3+^ ions in the KF-GdF_3_ host matrix class is attractive for multifunctional advanced materials: Yang et al. reported the magnetic and UC properties of Yb^3+^,Er^3+^,Ho^3+^,Tm^3+^: KGdF_4_ nanocrystals (Yang et al., 2010), whilst Kodama and Watanabe studied the visible quantum cutting based on downconversion in Eu^3+^: KGd_3_F_10_ crystals (Kodama and Watanabe, 2004).

Concerning thermal sensing results on Er^3+^-doped fluorides, several materials such as NaYF_4_: Yb^3+^,Er^3+^ (Vetrone et al., 2010; Zhou et al., 2013; Gonçalves et al., 2021); Er^3+^, Yb^3+^ co-doped KY_3_F_10_, KY_7_F_22_, and YF_3_ (Assy et al., 2016); SrF_2_: Yb^3+^,Er^3+^ (Senthilselvan et al., 2019); and NaLuF_4_: Er^3+^,Yb^3+^, Ho^3+^ (Runowski et al., 2018) are reported in the literature. These systems display relative sensitivity values between 0.75 and 1.30% K^−1^ at 300 K, which illustrate their potential use as thermometric probes. Particularly, Peng *et al.* investigated the temperature sensing potential of glass ceramics embedded with cubic Er^3+^, Yb^3+^: KGd_3_F_10_ nanocrystals, but the dimensions of this glass ceramic material is not ideal for *in vivo* applications.

As far as we know, the preparation of monodisperse, upconverting Er^3+^, Yb^3+^: KGd_3_F_10_ NPs, their use for bioimaging in the NIR-II region, and the exploitation of single Er^3+^, Yb^3+^: KGd_3_F_10_ NPs in nanothermometry have not been reported. Here, we have prepared Er^3+^, Yb^3+^: KGd_3_F_10_ NPs by the EDTA-assisted hydrothermal route to obtain monodisperse NPs with good biocompatibility. EDTA acts both as a surfactant and a chelating agent, enabling control of NP growth and aggregation via the hydrothermal methodology. We will describe the UC mechanisms involved in the NIR excitation of Er^3+^, Yb^3+^: KGd_3_F_10_ NPs at 980 and 1,550 nm and the luminescent thermometry measurements conducted on individual NPs.

## Materials and Methods

### Materials

Er_2_O_3_ (99.9%), Yb_2_O_3_ (99.9%), Gd(NO_3_)_3_•6H_2_O (99.9%), and anhydrous NH_4_F (≥98%) were purchased from Sigma Aldrich. KCl (99%) and HNO_3_ (65%) were acquired from Synth. Ethylenediaminetetraacetic acid (EDTA) was obtained from Alfa Aesar.

### Synthesis of Er^3+^, Yb^3+^ Co-Doped KGd_3_F_10_ Nanoparticles

The 5% Er^3+^, 20% Yb^3+^: KGd_3_F_10_ particles were synthesized by an ethylenediaminetetraacetic acid (EDTA)-assisted hydrothermal method. Erbium and ytterbium nitrate solutions were used as dopant precursors and were prepared by dissolving 10 mmol of the respective oxide in 100 ml of ∼0.1 mol.L^−1^ aqueous nitric acid solution. The concentrations of Er^3+^ and Yb^3+^ ions were 5 and 20 mol% in relation to the total RE^3+^ ions (1 mmol). Next, 0.75 mmol of Gd(NO_3_)_3_ was dissolved in 10.5 ml of the aqueous Er^3+^/Yb^3+^ solution, containing 0.05 and 0.2 mmol of Er^3+^ and Yb^3+^, respectively. After that, 15 ml of an aqueous solution containing 1 mmol of KCl was added to the resulting solution dropwise. Then, 15 ml of EDTA solution containing 1 mmol of EDTA was added to the solution containing the rare earth (RE^3+^) and K^+^ ions. Lastly, 10 mmol of NH_4_F dissolved in 15 ml of water was added to the final solution dropwise. After agitation for 15 min, the resulting milky solution was transferred to a Teflon bottle held in a stainless-steel autoclave and kept at 185°C for 15 h. After the reaction was complete, the final suspension was cooled to ambient conditions, and the particles were centrifuged. The wet powder was washed with distilled water and ethanol and dried in air at 80°C for 24 h.

### Characterization of the Materials

The morphology, size, and dispersion of the Er^3+^, Yb^3+^: KGd_3_F_10_ NPs were visualized with a JEOL JEM-100CX II transmission electron microscope (TEM) working at an accelerating voltage of 100 kV. The crystalline phase was determined by X-ray diffraction (XRD) analysis on a Siemens-Bruker D5005-AXS diffractometer operating with monochromatic CuKα1 radiation (wavelength at 154.18 pm); the diffractograms were acquired in the 2θ range between 10° and 90°, at a rate of 0.02°s^−1^. The Raman spectra were recorded on the LabRam Horiba Jobin-Yvon equipment. The spectra were obtained between 100 and 1,000 cm^−1^; a 633 nm laser as the excitation source, a 100 × lens for focusing the laser on the sample, a time acquisition of 40 s per point, and a grating of 600 gr/mm were employed. The NP dispersity in biological medium was investigated with a Dynamic Light Scattering (DLS) technique. 400 µg of NPs were dispersed in 2 ml of Dulbecco's Modified Eagle Medium (DMEM) enriched with 10% Fetal Bovine Serum (FBS). The DLS measurements were performed on a Zetasizer^®^ Nano ZS from Malvern PCS Instruments, United Kingdom. The UC spectra of the powder samples were recorded on a Fluorolog-3 Horiba Scientific (model FL3-22) equipped with double monochromators and a R928 Horiba photomultiplier; continuous wave (CW) CrystalLaser CL-2005 laser at 980 nm and CNI Laser (model MDL-N-1550-3W-18090494) at 1,550 nm were used as excitation sources. The NIR emission spectra were measured under excitation at 980 nm and registered with a Hamamatsu H10330-75 photomultiplier. The luminescence decay measurements were recorded with the same equipment by using a 450-W xenon pulsed lamp delivering pulses lasting 40 µs. A diluted dispersion was prepared for single NP studies, by suspending 0.01 g of Er^3+^, Yb^3+^: KGd_3_F_10_ NPs in 1 ml of isopropyl alcohol. Sonication for 5 min was carried out right before spin-coating of 10 µl of the dispersion on a glass coverslip (Menzel-Gläser #1) at 4,800 rpm for 20 s. For the optical studies on single NPs, a narrow-band (*δν*∼ 300 kHz) tunable diode laser (New Focus TLD 6900) tuned to 980 nm was sent to a homemade inverted microscope with microscope objective (×100, NA = 1.25), which focused the excitation beam on a single NP placed right at the focal spot by a 3-axis piezo stage. A calibrated heating device allowed for local temperature to be controlled over the investigated single NP. The luminescence was collected by the same objective and directed to a spectrometer or an avalanche photodiode with a flip mirror. The laser power pump was changed by using different combinations of neutral density filters with various optical densities. Details of the optical setup and the local temperature control system can be found in a previous work (Galvão et al., 2021a).

## Results and Discussion

### Structural and Morphological Properties

**FIGURE 1:**
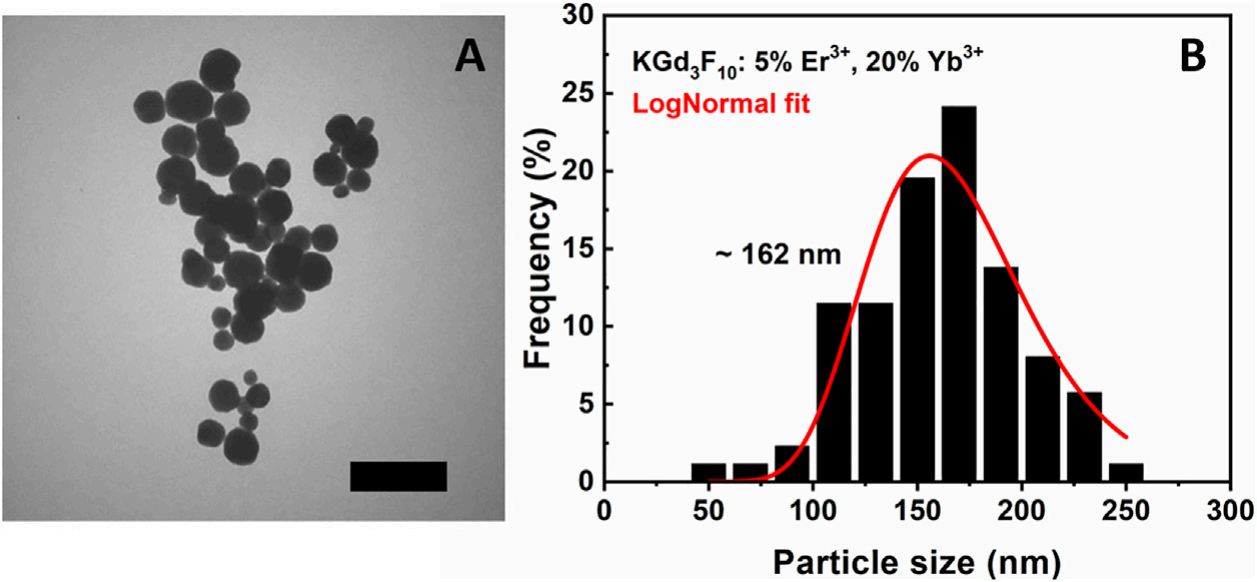
TEM image (2.5 µm × 2.5 µm) with scale bar of 500 nm **(A)** and histogram with log normal fit (red line) **(B)** of the 5% Er^3+^, 20% Yb^3+^: KGd_3_F_10_ particles. The peak of the log normal distribution occurs at ∼162 nm.

We synthesized the 5% Er^3+^, 20% Yb^3+^: KGd_3_F_10_ particles by the EDTA-assisted hydrothermal method adapted from (Lin et al., 2015). Figure 1 illustrates the TEM micrograph of the Er^3+^, Yb^3+^: KGd_3_F_10_ particles and the respective histogram.

Figure 1A shows well dispersed, spherical NPs with smooth surfaces. As mentioned previously, the use of EDTA reactant provides higher dispersity, as confirmed by the TEM image (Sun et al., 2007). The respective histogram reveals particle diameter distribution from 50 to 250 nm, with average particle size around 162 nm and standard deviation around ± 41 nm according to a log normal fit.

**FIGURE 2:**
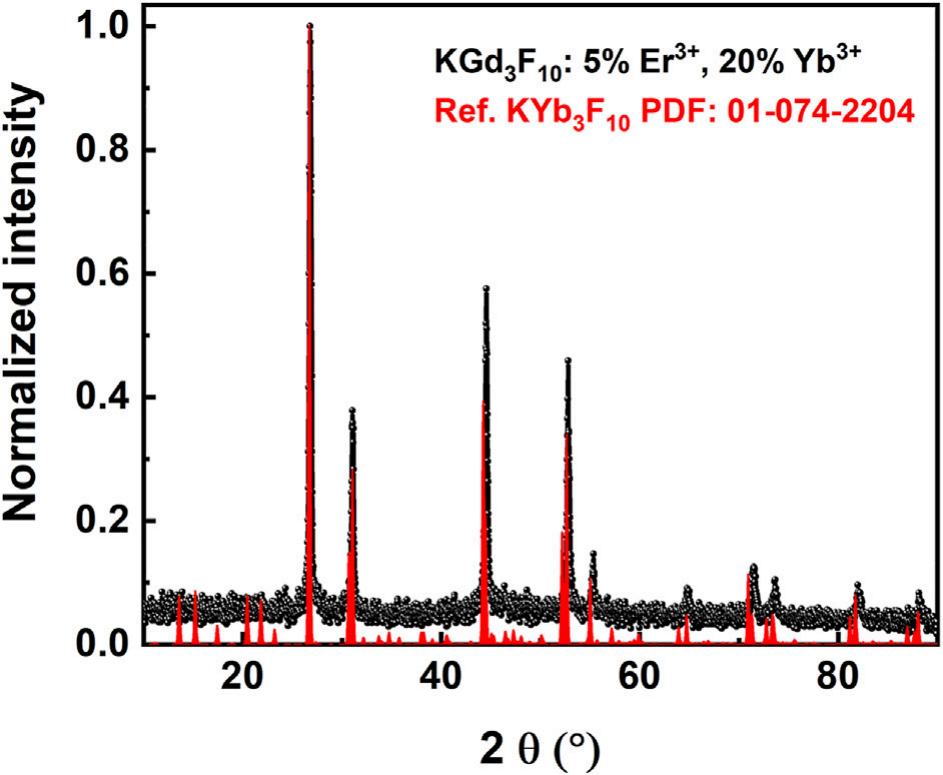
XRD pattern of the 5% Er^3+^, 20% Yb^3+^: KGd_3_F_10_ particles (black circles) and reference pattern of KYb_3_F_10_ provided by the ICSD online database (red vertical lines).

NPs with a single crystalline phase were formed through this synthetic route. Figure 2 displays the XRD pattern of the upconverting Er^3+^, Yb^3+^: KGd_3_F_10_ particles, which revealed reflection peaks that coincided with the reflection peaks attributed to the cubic KYb_3_F_10_ phase with space group Fm-3m (No. PDF: 01-074-2204), suggesting formation of the cubic phase of the KGd_3_F_10_ structure. The intense peaks indicated that hydrothermal synthesis gave highly crystalline particles, which dismissed the need for further thermal treatment. This is a crucial point in the synthesis of nanoparticulate materials given that thermal treatment after the use of high temperatures promotes agglomeration through sintering of the grains.

Raman vibrational spectroscopy was used to further elucidate the structural properties of the KGd_3_F_10_ fluoride. An unusual spectrum for a solid containing a single, very crystalline phase was observed with significant broadening, revealing specific structural aspects of the NPs.

**FIGURE 3:**
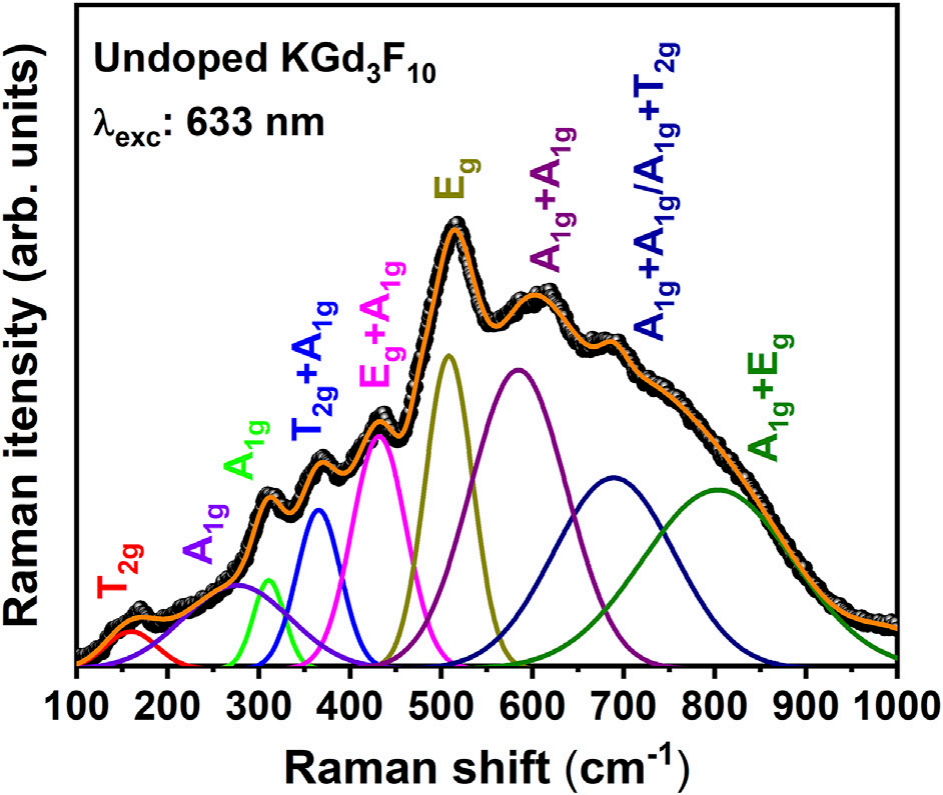
Deconvolution of the Raman spectrum of the undoped KGd_3_F_10_ NP powder performed at room temperature under excitation at 633 nm.

Figure 3 depicts the Raman spectrum of the undoped KGd_3_F_10_ NPs powder recorded under excitation at 633 nm and the respective deconvolution. The spectrum displayed a broadband in the low-frequency region from 100 to 800 cm^−1^ and resembled the spectrum reported by Serna-Gallén et al. for microstructured undoped KY_3_F_10_ cubic phosphors (Serna-Gallén et al., 2021). We assigned the deconvoluted KGd_3_F_10_ Raman bands according to the KY_3_F_10_ cubic phase, which presents the fluorite structure belonging to the Fm3m (Oh5) space group with two structural units per unit cell, with [KY_3_F_8_^(1)^]^2+^ and [KY_3_F_12_^(2)^]^2-^ ionic groups.

According to the group theory and irreducible representations of the O_h_ point group for cubic KY_3_F_10_, the irreducible representation for the Raman-active modes is Γ_Raman_ = 3A_1g_+4E_g_ +6T_2g_ (Mortier et al., 1991). The Raman lines reported by Serna-Gallén *et al.* for KY_3_F_10_ cubic phosphors and the Raman spectrum obtained in this work are broader compared to the Raman spectra of single KY_3_F_10_ crystals reported by Mortier et al. (1991). The Raman spectrum broadening observed here could be related to the presence of defects and distortions in the cubic crystalline lattice of these NPs, where low-symmetry phases co-exist with distorted octahedra, and to the combination of bands and second-order overtones. Overtones are always Raman-allowed, and the broad Raman bands are Raman-active according to the direct product of the irreducible representations associated with the respective phonon vibration modes (Toulouse et al*.*, 2019).

**FIGURE 4:**
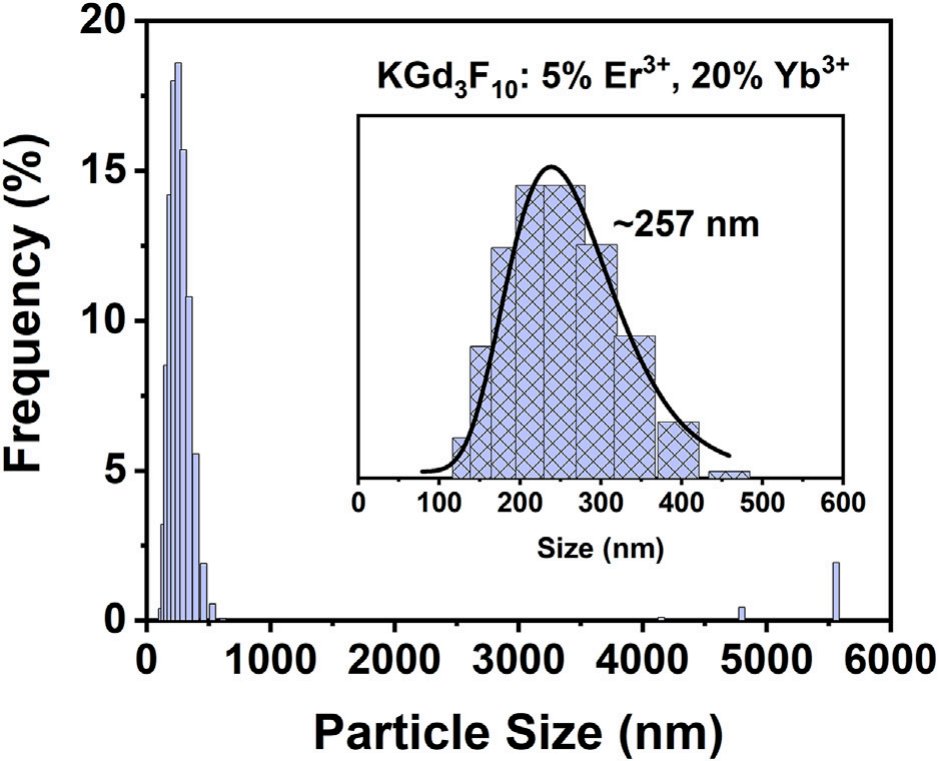
Hydrodynamic diameter distribution of the 5% Er^3+^, 20% Yb^3+^: KGd_3_F_10_ NPs dispersed in DMEM medium with 10% FBS obtained by DLS technique.

We dispersed the Er^3+^, Yb^3+^: KGd_3_F_10_ particles in DMEM enriched with 10 % FBS to evaluate their dispersity level in biological medium by means of DLS. Figure 4 shows excellent dispersion of the Er^3+^, Yb^3+^: KGd_3_F_10_ NPs in a biological medium if we consider the high population of species with sizes varying between 125 and 475 nm and the presence of few aggregates with sizes ranging between 4,700 and 5,550 nm. According to the log normal adjustment, such major hydrodynamic size distribution was centered at approximately 257 nm. The polydispersity index of this distribution is 0.316. This indicated that the aggregation index was low if we bear in mind that the average size obtained by TEM measurements was around 162 nm. The DLS characterization suggested that the system has high dispersity level in biological medium and is promising for *in vivo* applications.

### Powder Optical Spectroscopy

**FIGURE 5:**
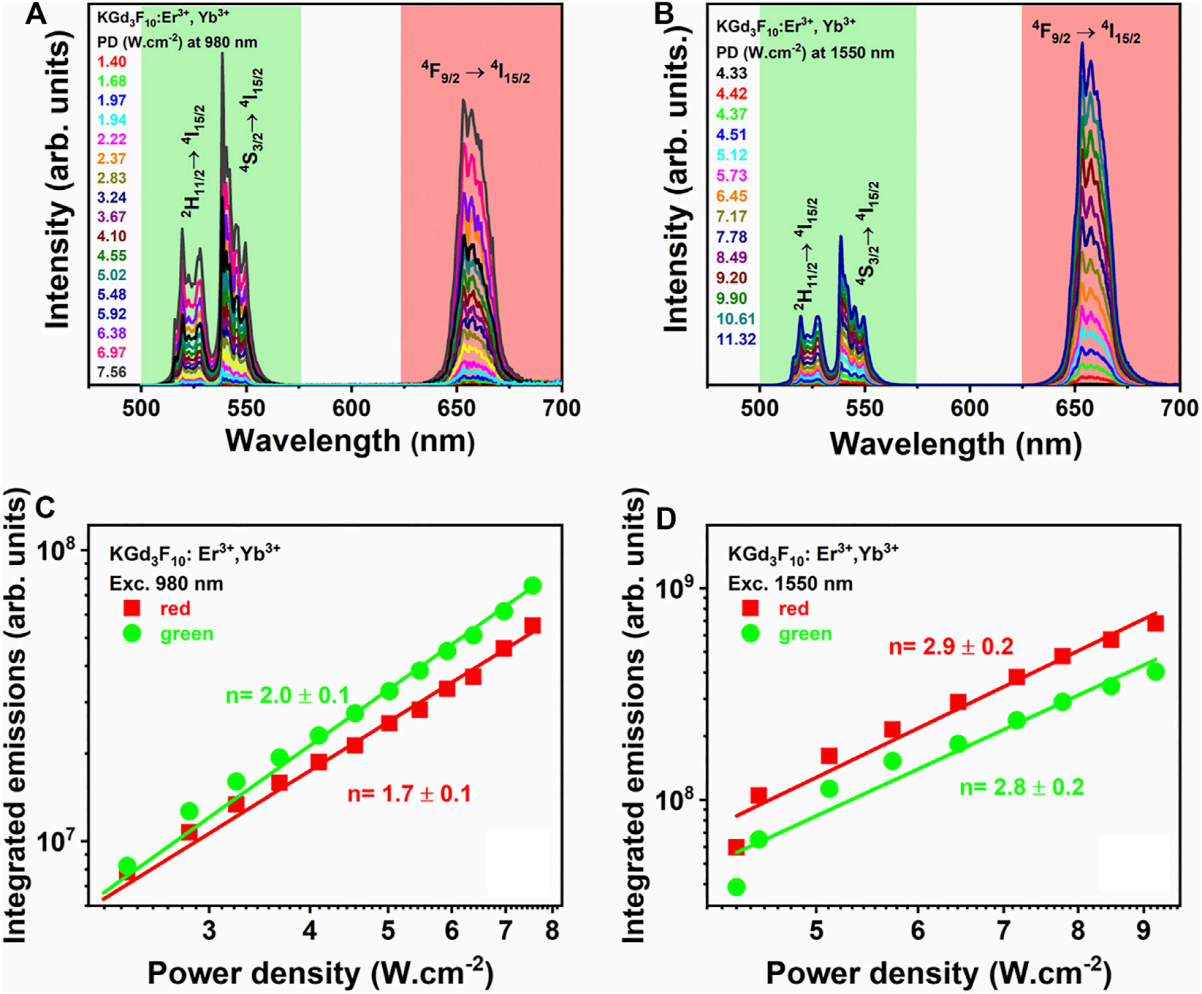
UC emission spectra of the 5% Er^3+^, 20% Yb^3+^: KGd_3_F_10_ powder obtained by varying the excitation powers at 980 **(A)** and 1,550 nm **(B)**, and the respective log-log plot of the areas under the UC spectra of the green and red emission bands as a function of the 980- and 1550 nm excitation power densities **(C)** and **(D)**, respectively.

We started the optical characterization of the synthesized NPs by accomplishing UC measurements, initially performed on the Er^3+^, Yb^3+^: KGd_3_F_10_ powder under CW excitation in the infrared region at 980 and 1,550 nm. Figure 5 shows the UC spectra as a function of the excitation power at both excitation wavelengths.

The emission spectra displayed the green and red UC emission bands assigned to the ^2^H_11/2_ → ^4^I_15/2_ (*λ* = 512–537 nm), ^4^S_3/2_ → ^4^I_15/2_ (*λ* = 540–560 nm), and ^4^F_9/2_ → ^4^I_15/2_ (*λ* = 645–680 nm) transitions of Er^3+^ ions (Figures 5A,B). The UC mechanisms for generating the green and red emissions can be characterized, among other aspects, by evaluating the area A of the bands in the fluorescence spectra corresponding to both transitions as a function of the excitation power density PD (in a log-log graph), shown in Figures 5C,D. The power law expressed by A ∝ PD ^n^ gives the number n of photons involved in the UC process. We observed n values around 2.0 and 1.7 for the green and red emissions obtained under excitation at 980 nm, respectively (Figure 5C), which indicated that two photons (at 980 nm) were absorbed for the respective UC emissions to occur. Two-photon absorption mechanisms can explain the green and red UC emissions under NIR excitation, mainly the Excited State Absorption (ESA) and the Energy Transfer Upconversion (ETU) processes (Auzel, 2004).

**FIGURE 6:**
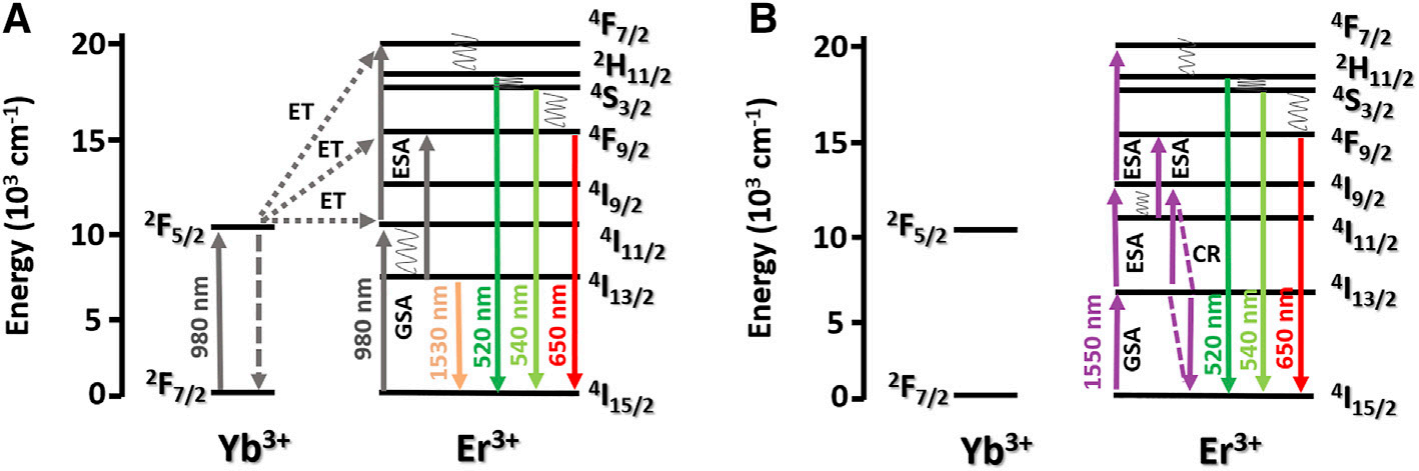
Simplified energy-level diagram of the Er^3+^ and Yb^3+^ ions and the proposed mechanisms, under excitations at 980 **(A)** and 1,550 nm **(B)**. Upward and downwards solid arrows indicate photon absorption and emission, respectively. Dashed arrows stand for cross-relaxation processes.

In the ESA mechanism, one Er^3+^ ion is excited to the ^4^I_11/2_ level by ground state absorption (GSA), which is followed by absorption of a second photon of the same energy, bringing the excited ion to the higher ^4^F_7/2_ energy level (Figure 6A). The excited ions decay non-radiatively to the ^2^H_11/2_ and ^4^S_3/2_ emitting levels, emitting in the green, and to the ^4^F_9/2_ emitting level, emitting in the red due to the fast multi-phonon non-radiative decay from the ^4^F_7/2_ level to the ^2^H_11/2_ level. As for the ETU process, there are two possible mechanisms involving only Er^3+^/Er^3+^ and Er^3+/^Yb^3+^ pairs. For the first pair, two Er^3+^ ions can be excited to the ^4^I_11/2_ level by GSA and exchange energy, and one of them decays to the ground state, while the other one is excited to the ^4^F_7/2_ level. In the second ETU process, one Er^3+^ ion is excited to the ^4^I_11/2_ level and one Yb^3+^ ion is excited to the ^2^F_5/2_ level via GSA. Then, Yb^3+^ ions transfer energy to Er^3+^ ions, which is excited to the ^4^F_7/2_ level. Taking into account that Yb^3+^ ions have higher absorption cross-section than Er^3+^ ions, the former strongly absorb the NIR excitation photons, populating the ^2^F_5/2_ level of Yb^3+^ ions, which in turn transfer energy to Er^3+^ ions, populating the ^4^F_7/2_ energy level of Er^3+^ ions. In both situations, Er^3+^ ions relax non-radiatively to the ^2^H_11/2_, ^4^S_3/2_, and ^4^F_9/2_ levels, emitting in the green and red spectral regions, respectively (Auzel, 2004). In both processes, Er^3+^ ions excited to the ^4^I_11/2_ level by GSA can also decay non-radiatively to the ^4^I_13/2_ level, which is followed by absorption of a second photon, populating the ^4^F_9/2_ level and causing the ^4^F_9/2_ → ^4^I_15/2_ electronic transition.

As previously discussed, 1,550 nm wavelength is placed on the NIR II-B biological window which shows deeper penetrability in biological tissues in comparison to visible spectral range excitation (Hemmer et al., 2013). In this work, the mechanisms of UC through excitation as 1,550 nm were also evaluated, opening other excitation possibilities for *in vivo* applications, due to the lower light scattering coefficient compared with 980-nm excitation (Wang et al., 2018; Yamanaka et al., 2019; Pham et al., 2021).

For the UC emission spectra obtained under excitation at 1,550 nm (Figure 5B), the red emission was more intense than the green emission, in contrast with the UC emission obtained under excitation at 980 nm. This behavior demonstrated that the excitation wavelengths influenced the UC mechanisms. The UC emission intensities as a function of the excitation powers presented n values around 2.8 and 2.9 for the green and red emissions, respectively. These n values indicated that, under excitation at 1,550 nm, three photons were absorbed to generate these UC emissions (Figure 5D). The UC mechanisms proposed for the 1550 nm photon absorption involve Er^3+^ ions only (Figure 6B). Initially, the first 1550 nm photon is absorbed, exciting Er^3+^ ions to the ^4^I_13/2_ level by GSA. This is followed by absorption of a second photon, bringing the excited ions to the higher ^4^I_9/2_ energy level. In addition, the ^4^I_9/2_ excited level can be populated by cross-relaxation (CR) process (^4^I_13/2_ + ^4^I_13/2_→^4^I_9/2_ + ^4^I_15/2_). Absorption of the third photon occurs from the ^4^I_9/2_ energy level, to populate the ^4^F_7/2_ excited level, which decays non-radiatively to the lower ^2^H_11/2_ and ^4^S_3/2_ levels. The latter excited levels are responsible for the green emission, assigned to the ^2^H_11/2_ → ^4^I_15/2_ (*λ* = 512–537 nm) and ^4^S_3/2_ → ^4^I_15/2_ (*λ* = 540–560 nm) transitions of Er^3+^ ions. The second UC mechanism involving 1550-nm photon absorption considers the non-radiative depopulation from the ^4^I_9/2_ excited level to the ^4^I_11/2_ level (by multi-phonon relaxation), followed by absorption of the third photon to the ^4^F_9/2_ level. From this level, Er^3+^ ions decay radiatively to the ^4^I_15/2_ ground level, emitting red radiation. The last UC mechanism is dominant for 1550 nm photon absorption: the red emission is more intense than the green emission, so the ^4^I_11/2_ level is more populated compared to the ^4^I_9/2_ excited level of Er^3+^ ions.

**FIGURE 7:**
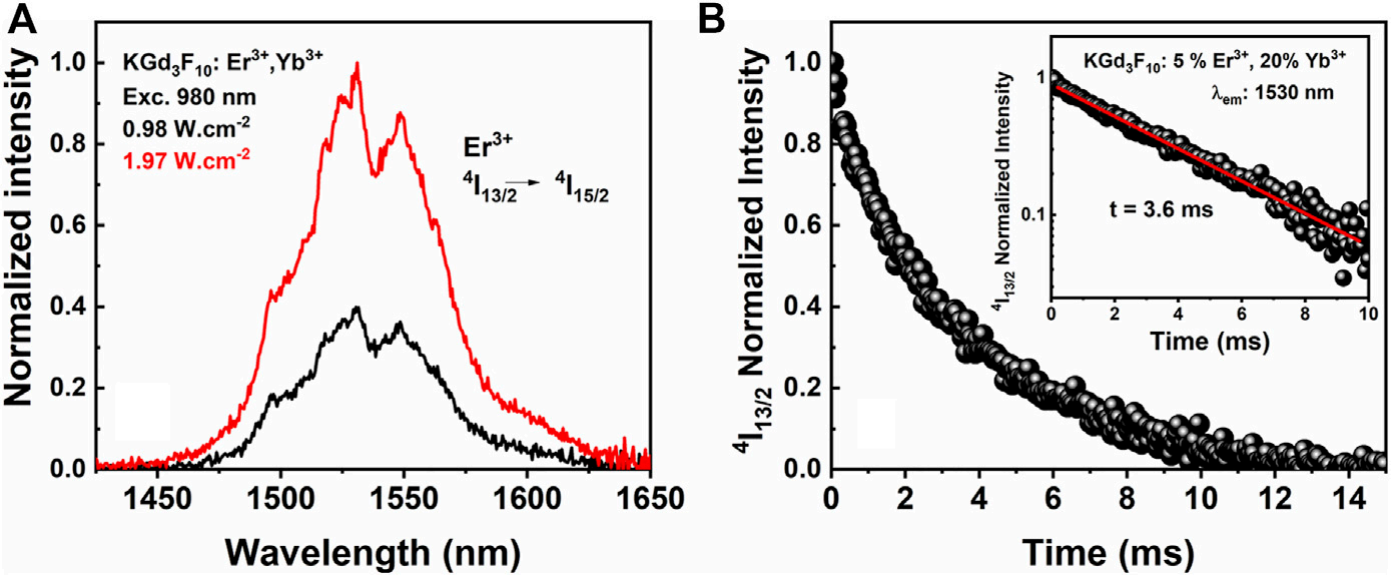
Photoluminescence spectra in the 1,550 nm range assigned to the ^4^I_13/2_ →^4^I_15/2_ transition upon excitation at 980 nm for different power densities **(A)** and decay curve from the ^4^I_13/2_ metastable level under excitation at 273 nm **(B)** of the 5% Er^3+^, 20% Yb^3+^: KGd_3_F_10_ NP powder.

Apart from the UC emissions, the NPs synthesized herein also presented an emission band in the NIR-II region (Figure 7A).

We recorded broadband emission at low excitation power density excitation at 980 nm, with the maximum intensity centered at 1,530 nm. We assigned the strong NIR emission to the ^4^I_13/2_→^4^I_15/2_ transition of Er^3+^ ions, which occurs through excitation of Yb^3+^ ions to their ^2^F_5/2_ excited level, followed by energy transfer to the ^4^I_11/2_ excited level of Er^3+^ ions. The electron in the ^4^I_11/2_ level of Er^3+^ ions decayed non-radiatively to the lowest ^4^I_13/2_ excited level of these same ions, leading to the strong NIR-II emission centered at 1,530 nm. Compared to the NIR-I imaging window (700–900 nm), the NIR-II window presents improved performance due to reduced absorption, photon scattering, and tissue autofluorescence (Venkatachalam et al., 2013; Ding et al*.*, 2018; Xu et al., 2019). Figure 7B shows the NIR luminescence decay curve of the ^4^I_13/2_ excited level of Er^3+^ ions from the UV excitation in the Gd^3+^ host, at 273 nm. It was possible to fit the decay by single exponential adjustment. The ^4^I_13/2_ lifetime was 3.6 ms, which was close to the values between 3.22 and 3.47 ms reported by Karthi *et al.* for Er^3+^-doped fluoroapatite NPs (Karthi et al., 2016). However, Fan and co-authors found Er^3+ 4^I_13/2_ lifetimes ranging from 1.25 to 20.9 ms for hexagonal NaGdF_4_@NaGdF_4_:Yb,Er@ NaYF_4_:Yb@NaNdF_4_:Yb core-shell systems (Fan et al., 2018). The authors attributed the longer excited state lifetimes to the thickness of the Yb^3+^-doped shell, which increased from 0 to 7 nm. These core-shell NPs had diameters smaller than 30 nm, so the surface-to-volume ratio played a crucial role in the luminescence lifetimes, which made protection of the Er^3+^ surface ions necessary. In our case, the surface-to-volume ratio did not influence the Er^3+^ excited state lifetimes significantly because the average particle size was around 160 nm.

Overall, monodisperse Er^3+^, Yb^3+^ co-doped KGd_3_F_10_ NPs are promising candidates for NIR-II biomedical imaging, medical therapies, and optical thermometry because they present high dispersity in biological medium, visible and NIR emissions, with long ^4^I_13/2_ lifetimes (NIR II region). Despite these preliminary and promising results, further studies are necessary to evaluate the cellular toxicity of this matrix for application in theranostics, where they can act as biomarkers and drug activators of some photobiological response as a coadjuvant therapy for cancer treatment. The cellular nontoxicity of cubic Er^3+^, Yb^3+^ co-doped KY_3_F_10_ NPs to glioblastoma multiforme (GBM) cells has been previously investigated by our group, and we obtained viability values close to 100% for this similar compound (de Oliveira Lima et al., 2021). Designing suitable core-shell structures is still an essential task to improve the optical properties of this lanthanide-based system. Here, we tried to characterize a new optical nanothermometer, to establish a new path for KGd_3_F_10_ system applications. In the future, there still will be a great demand for the development of novel upconverting nanoprobes.

Next, we observed emissions at low excitation power densities at 980 nm with relatively long luminescence lifetimes, which could open the possibility of using these upconverting NPs for NIR *in vivo* imaging.

### Single Nanoparticles Optical Spectroscopy

**FIGURE 8:**
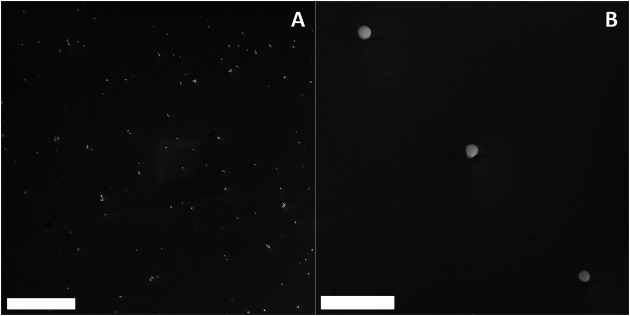
Large-scale (46.4 µm × 46.4 µm) Scanning Electron Microscopy (SEM) of upconverting KGd_3_F_10_ NPs deposited on a coverslip after spin-coating a dilute dispersion of NPs on it (scale bar: 10 µm) **(A)**. Small-scale scanning (4.64 µm × 4.64 µm), where it is possible to choose single NPs located far away from any other NP (at least 3.2 µm, about 10× the theoretical resolution of the optical system) to perform the single NP measurements (scale bar: 1 µm) **(B)**.

We carried out scanning electron microscopy (SEM) to evaluate particle deposition on a glass-coverslip for single particle optical spectroscopy investigation (Figure 8).

We investigated the structural, morphological, and optical properties of the Er^3+^, Yb^3+^: KGd_3_F_10_ for better understanding of the nature of the KGd_3_F_10_ NPs. For the optical experiments on single NPs, we suspended the powder sample in isopropyl alcohol for NP deposition, so that we would have access to individual NPs. The SEM images showed the excellent dispersion of the Er^3+^, Yb^3+^: KGd_3_F_10_ particles deposited on the glass-coverslip substrate by the spin-coating technique. Additionally, the SEM micrographs agreed with the particle diameter analysis from the TEM picture, showing dispersed objects with diameter sizes ranging from 125 to 140 nm.

**FIGURE 9:**
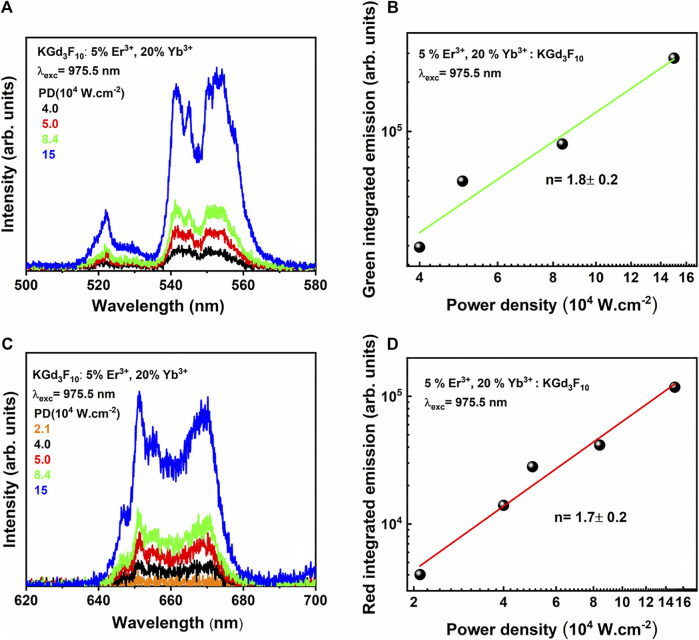
UC green **(A)** and red **(C)** emission spectra of a single Er^3+^, Yb^3+^: KGd_3_F_10_ NP upon varying the power densities at 975.5 nm and log-log plot of the UC spectrum area of the green **(B)** and red **(D)** emissions as a function of the power densities of the same individual NP.

After we ensured that single NPs were dispersed on the glass substrate, we performed UC measurements as a function of the pump power at 975.5 nm for several individual particles. Figure 9 shows the UC green and red emission spectra obtained by varying the power density for an isolated particle, as well as the respective dependence of the UC emission intensities on the pump power density.

Figure 9A presents the UC emission spectra of a single NP, measured between 500 and 580 nm as a function of the CW excitation power at 975.5 nm, under power densities ranging from 2.1 to 15.10^4^ W cm^−2^. We ascribed the emission bands corresponding to the green emission to the ^2^H_11/2_ → ^4^I_15/2_ (centered at 525 nm) and ^4^S_3/2_ → ^4^I_15/2_ transitions (ranging from 540 to 570 nm) of Er^3+^ ions. Concerning the red UC emission, Figure 9C shows the corresponding spectra measured specifically between 625 and 700 nm of the same Er^3+^, Yb^3+^: KGd_3_F_10_ single NP with excitation fixed at 975.5 nm and several power densities. To evaluate how the UC emission intensity varied with the excitation power, we used neutral density filters in the excitation beam path. We obtained n values around 1.8 ± 0.2 and 1.7 ± 0.2 for the green and red emissions, respectively (Figures 9B,D), indicating that two photons had to be absorbed for the respective UC emissions to occur. As discussed previously for the powder UC measurements, the ESA and ETU two-photon absorption mechanisms account for the observed UC emissions. Comparison of both analyses, UC measurements on the isolated NP and the NP ensemble, clearly showed that the saturation regime was not reached at used power pump in the case of single NPs, as depicted in the graphs in Figures 9B,D.

### Single Nanoparticles Luminescence Intensity Ratio Thermometry

**FIGURE 10:**
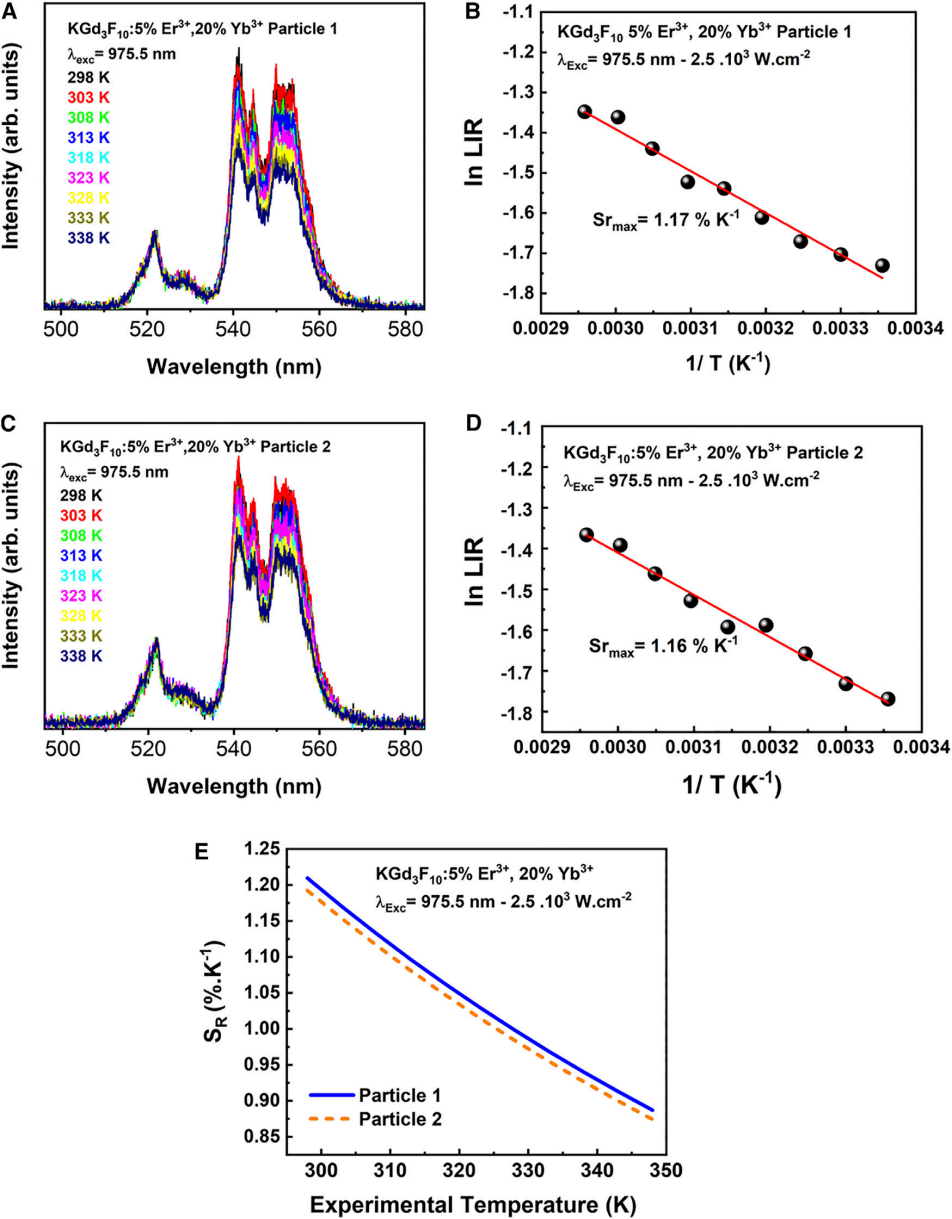
Temperature-dependent behavior of the UC spectra from 298 to 338 K under excitation at 975.5 nm with 2.5 .10^3^W cm^−2^ power density **(A, C)**, relative sensitivities obtained in the green range, under excitation at 975.5 nm **(B)** and **(D)**, for the two single Er^3+^, Yb^3+^: KGd_3_F_10_ particles. Thermometric parameter S_R_ versus experimental temperature **(E)** for two single Er^3+^, Yb^3+^: KGd_3_F_10_ NPs.

After we performed UC experiments on an individual dried NP, we investigated thermal sensitivities by using the LIR technique. Figures 10A,C shows the green region of the UC emitting spectra of two different Er^3+^, Yb^3+^: KGd_3_F_10_ NPs as a function of temperature from 298 to 338 K, under the excitation at 975.5 and power density of 2.5 .10^3^W cm^−2^. In the UC emission spectra, the emission intensities related to the ^4^S_3/2_ → ^4^I_15/2_ transition (*λ* = 540–560 nm) varied significantly with increasing temperature, while the intensities of the ^2^H_11/2_ → ^4^I_15/2_ transition (*λ* = 512–537 nm) remained practically constant.

Figures 10B,D show the LIR behavior of two single particles at temperatures ranging between 298 and 338 K (*R* ^2^ = 0.97 for particle A and *R* ^2^ = 0.98 for particle B, see discussion below). The energy gap between the ^2^H_11/2_ and ^4^S_3/2_ levels was small enough for thermal equilibrium to be almost reached.

The intensity of the radiation emitted by the ^2^H_11/2_ and ^4^S_3/2_ thermally coupled levels is proportional to their relative populations. These are described by the Boltzmann population distribution law. Thus, LIR can be related to the intensity of the radiation emitted in the two green UC bands as (Menezes and De Araújo, 2015):

$$LIR=\;\frac{I_{525\;nm}}{I_{555\;nm}}=A\exp\left(\frac{-E_{21}}{kT}\right)\;$$ (1)

where *E*_*21*_ is the energy gap between both thermally coupled energy levels, and *k* is the Boltzmann constant. The constant *A* fundamentally depends on the degeneracies (2J + 1) of both energy levels, the total spontaneous emission rates, and the angular frequencies of the |2> → |0> and |1> → |0 > transitions associated with the ^2^H_11/2_ → ^4^I_15/2_ and ^4^S_3/2_ → ^4^I_15/2_ transitions of Er^3+^ ions (Shinn et al., 1983; Brites et al., 2016; Balabhadra et al., 2017).

In this sense, to define ratiometric property, the Boltzmann population behavior must settle just above room temperature (Suta et al., 2020). Here, we demonstrate that a Boltzmann type distribution was observed. Equation 1 can be written as follows:

$$\ln LIR=\;\beta \;-\;\frac{\alpha }{T}\;$$ (2)

where *β* = ln (A) and *α* = $\frac{E_{21}}{k}$. In this way, *β* specifies a dimensionless parameter, and *α* has dimensions of temperature and indicates the effective energy separation between the thermally coupled levels for the LIR.

Thermal sensitivity is the rate at which the intensity ratio changes with temperature (Menezes and De Araújo, 2015), so

$$S=\frac{dLIR}{dT}=LIR\;\frac{\alpha }{T^{2}}$$ (3)

Nevertheless, to evaluate the performance of the luminescence thermometer, the relative sensitivity is an important figure of merit reported in several materials (Ananias et al*.*, 2018; Runowski et al*.*, 2018):

$$S_{R}=\frac{S}{LIR}=\frac{\alpha }{T^{2}}$$ (4)

We found maximum relative sensitivities of 1.17 ± 0.03 and 1.16 ± 0.03% K^−1^ for two different single particles at 298 K. Furthermore $S_{R}$, temperature accuracy *δT* is another parameter to describe the luminescence thermometer. It gives the uncertainty of a temperature measurement as a result of the smallest area increment that can be measured by LIR. *δT* is given by (Galvão et al., 2021a):

$$\text{δT}=\frac{\text{δ}\left(\text{LIR}\right)}{S_{R}\left(LIR\right)}$$ (5)

Where *δ(LIR)/LIR* is the relative error of temperature measurement parameter *LIR*, which value in our case is 0.14%. Therefore, *δT* ≈ 0.1 K, being typical value for NPs-based sensors (Martín et al., 2011; Galvão et al., 2021b). Such$\;S_{R}$ values were close to some values reported in the literature for the ^2^H_11/2_ → ^4^I_15/2_ and ^4^S_3/2_ → ^4^I_15/2_ transitions of Er^3+^ ions. Peng *et al.* studied Er^3+^, Yb^3+^: KGd_3_F_10_ glass ceramics prepared via the melting-quench route followed by thermal treatment of 700°C for 2 h and obtained S_R_ around 1.35% K^−1^ at 303 K (Peng et al., 2018). According to Assy and collaborators (Assy et al., 2016), Er^3+^, Yb^3+^ co-doped KY_3_F_10_ particles prepared via the hydrothermal method at 180°C for 48 h presented relative thermal sensitivity around 1.30% K^−1^ at 300 K. Senthilselvan *et al.* reported 1.22% K^−1^ at 300 K for Er^3+^, Yb^3+^: SrF_2_ particles prepared by the EDTA-assisted hydrothermal method followed by heat treatment at 450°C for 3 h (Senthilselvan et al., 2019). Gonçalves *et al.* evaluated the thermal properties of Er^3+^, Yb^3+^co-doped *ß*-NaYF_4_ microcrystals prepared via the thermal decomposition method and obtained S_R_ value of 1.25% K^−1^ at 310 K (Gonçalves et al., 2021). Runowski and co-authors reported S_R_ of 0.75% K^−1^ at 300 K for *ß*-NaLuF_4_:Yb^3+^, Er^3+^, Ho^3+^ microcrystals obtained by the citric acid-assisted hydrothermal method (Runowski et al., 2018).

The differences between the S_R_ values reported for the fluorides in the cited references and our Er^3+^, Yb^3+^: KGd_3_F_10_ NPs were associated with variation in the *E*_*21*_ values. Therefore, the larger the separation between the *E*_*21*_ energy levels, the better the relative sensitivity of the respective material. RE^3+^ transitions involve 4f^n^ levels shielded by more external electronic sublevels, but their positions vary slightly in different hosts. Thus, the energy gap between both thermally coupled Er^3+^ energy levels can be related to the reactional and post-reactional material parameters, such as the type of synthesis, reaction times, and additional thermal treatment (Bettinelli et al., 2015; Brites et al., 2016). Li et al. reported that particle sizes and shapes influence the thermal sensitivities of NaYF_4_:Yb^3+^, Er^3+^ particles and showed that the surface-to-volume ratio affect the separation between the Er^3+^ energy levels (Li et al., 2013). These conditions strongly affect the host structure in terms of morphology, crystalline phase, and presence of strain and defects. Here, the Er^3+^, Yb^3+^: KGd_3_F_10_ NPs probably presented more structural defects than the systems prepared for longer reaction times and with additional annealing, as reported by Peng *et al.* for Er^3+^, Yb^3+^: KGd_3_F_10_ glass ceramics (Peng et al., 2018). It is worth remembering that our as-prepared NPs were well crystallized and dismissed the need for further post-thermal treatment, avoiding the performance of an annealing process that could promote NP aggregation as well as NP monodispersion in biological medium. The good crystallinity of our monodisperse as-prepared NPs comes from the chosen hydrothermal synthesis. This kind of method presents the advantages of fluoride preparation with controllable morphologies, sizes and architectures in a relatively simple and green way to produce a large amount of product (Byrappa and Adschiri, 2007; Komarneni et al., 2010). The EDTA-assisted hydrothermal synthesis allows the investigation of isolated particles, this single particle study would not have been possible if the NPs would be synthesized by the melting-quench route. These results support the idea that our material can be applied as a multifunctional platform and operate as a thermometer with high relative thermal sensitivity for biological applications, for instance.

As predicted by Eq. 4, Figure 10E shows that S_R_ decreased with increasing temperature. Assuming a quality requirement of $S_{R}$ ≥ 1.0% K^−1^, the ideal operational range for this system would be 298–323 K. Because it is important to explicit the behavior of $S_{R}$ as a function of the target temperature range to a point where the thermometric properties can be impaired, the thermometric properties of single Er^3+^, Yb^3+^: KGd_3_F_10_ NPs must be investigated to make this kind of material a multifunctional platform for biological applications, such as imaging, treatment, and thermal probe, where thermal sensitivity operates at temperature values befitting for living systems.

## Conclusion

We have successfully prepared highly crystalline Er^3+^, Yb^3+^: KGd_3_F_10_ NPs by the EDTA-assisted hydrothermal route. The DLS measurements and TEM/SEM micrographs revealed excellent particle dispersion: we achieved well-dispersed particles with average diameter around 162 nm. These highly colloidal luminescent NPs display strong green and red emission transitions under infrared excitation (at 980 and 1,550 nm). We attribute the two- and three-photon processes to the UC mechanisms for the powder sample under excitation at 980 and 1,550 nm. We observed strong NIR emission centered at 1,530 nm under low 980 nm power pump. When excited at 975.5 nm with a narrow-band laser, the individual particles present two-photon absorption related to the green and red emissions. The experimental results regarding nanothermometry with single NP level for the dry state showed sensitivities of 1.16 and 1.17% and accuracy of 0.1 K at 300 K for two single particles. The intense UC emissions and the good thermal sensitivity of the fluorescent single Er^3+^, Yb^3+^: KGd_3_F_10_ particles indicate their potential application as bioprobes and accurate nanothermometers in the thermal range of biological interest. To the best of our knowledge, this is the first report of promising thermometry results for single KGd_3_F_10_ particles.

## Data Availability

The original contribution presented in the study is included in this paper, further inquiries can be directs to the corresponding author.
